# Whole genome HBV deletion profiles and the accumulation of preS deletion mutant during antiviral treatment

**DOI:** 10.1186/1471-2180-12-307

**Published:** 2012-12-28

**Authors:** Dake Zhang, Peiling Dong, Ke Zhang, Libin Deng, Christian Bach, Wei Chen, Feifei Li, Ulrike Protzer, Huiguo Ding, Changqing Zeng

**Affiliations:** 1 Laboratory of Disease Genomics and Individualized Medicine, Beijing Institute of Genomics, Chinese Academy of Sciences, Beijing, China; 2 Beijing Youan Hospital, Capital Medical University, Beijing, China; 3 Institute of Virology, Technische Universität München / Helmholtz Zentrum München - German Research Center for Environmental Health, München, Germany; 4 Basic Medical Institute of Nanchang University, Nanchang, China; 5 No.7 Beitucheng West Road, Chaoyang District, Beijing, 100029, China; 6 No.8 Xitoutiao, You An Men, Beijing, 100069, China

**Keywords:** HBV, Deletion, PreS, Chronic hepatitis, Antiviral therapy, Nucleotide analog

## Abstract

**Background:**

Hepatitis B virus (HBV), because of its error-prone viral polymerase, has a high mutation rate leading to widespread substitutions, deletions, and insertions in the HBV genome. Deletions may significantly change viral biological features complicating the progression of liver diseases. However, the clinical conditions correlating to the accumulation of deleted mutants remain unclear. In this study, we explored HBV deletion patterns and their association with disease status and antiviral treatment by performing whole genome sequencing on samples from 51 hepatitis B patients and by monitoring changes in deletion variants during treatment. Clone sequencing was used to analyze preS regions in another cohort of 52 patients.

**Results:**

Among the core, preS, and basic core promoter (BCP) deletion hotspots, we identified preS to have the highest frequency and the most complex deletion pattern using whole genome sequencing. Further clone sequencing analysis on preS identified 70 deletions which were classified into 4 types, the most common being preS2. Also, in contrast to the core and BCP regions, most preS deletions were in-frame. Most deletions interrupted viral surface epitopes, and are possibly involved in evading immuno-surveillance. Among various clinical factors examined, logistic regression showed that antiviral medication affected the accumulation of deletion mutants (OR = 6.81, 95% CI = 1.296 ~ 35.817, P = 0.023). In chronic carriers of the virus, and individuals with chronic hepatitis, the deletion rate was significantly higher in the antiviral treatment group (Fisher exact test, P = 0.007). Particularly, preS2 deletions were associated with the usage of nucleos(t)ide analog therapy (Fisher exact test, P = 0.023). Dynamic increases in preS1 or preS2 deletions were also observed in quasispecies from samples taken from patients before and after three months of ADV therapy. In vitro experiments demonstrated that preS2 deletions alone were not responsible for antiviral resistance, implying the coordination between wild type and mutant strains during viral survival and disease development.

**Conclusions:**

We present the HBV deletion distribution patterns and preS deletion substructures in viral genomes that are prevalent in northern China. The accumulation of preS deletion mutants during nucleos(t)ide analog therapy may be due to viral escape from host immuno-surveillance*.*

## Background

The high mutation rate of the hepatitis B virus (HBV) is responsible for diverse viral mutants that are resistant to antiviral therapies [1,2]. In addition to single base substitutions, a number of deletion mutations have also been reported. Deletion hotspots include precore/core genes, the preS region, and the region of X gene overlapped with basic core promoter (BCP) [3,4]. Deletions are believed to be associated with the progression of viral hepatitis. Coexistence of wild type HBV (*wt*), relative to deleted sequences, and mutants with deletions in the C gene has been shown to enhance viral replication, which may be mediated by the coordination of *wt* and viral strains during encapsidation or reverse transcription [5]. Core deletions have frequently been detected before seroconversion to anti-HBe [6]. Mutations in codons 130 and 131 of the X gene, with deletions of nucleotides 1762 and 1764 respectively, were reported to be common in hepatocellular carcinoma (HCC) patients [7,8]. Furthermore, preS deletion mutants produce truncated HBV surface proteins (large and middle HBsAg (L- and M-HBsAg)), which accumulate in the endoplasmic reticulum (ER). This has been shown to increase ER pressure, which promotes the expression of cyclin A and the host apoptosis suppressor cyclooxygenase-2 [9,10]. These findings have raised concerns regarding preS deletions as a risk factor for hepatocarcinogenesis [11-14]. Despite certain complex viral deletion patterns revealed in previous studies [4], we do not yet fully understand the pattern of these deletions and their correlation to clinical factors.

Many deletions interrupt epitopes of viral proteins recognized by T- or B-cells. For instance, the internal deletion around aa 81–136 of core antigen damages a T-cell epitope [15,16]. PreS truncations were reported to be associated with the loss of T- and B-epitopes that were able to elicit host protective immune responses [17,18]. In addition, deletions that disrupt the X gene may lead to low expression of HBcAg as observed by the lack of HBc antibody in patients [19-21]. Hence, HBV deletions are speculated to assist viruses in the evasion of immunologic surveillance. Additionally, some deletion mutations are more frequently observed in certain clinical conditions. For instance, an nt 1770–1777 deletion in the X gene of HBV was detected in many serologically non-B and non-C patients [19,20]. However, most studies only investigate one or two deletion hotspots, thus lacking the complete view of deletion patterns provided by whole genome sequence analyses.

In this study, we describe the distribution of prevailing deletions from 51 patient genomes and 70 genome fragments with preS deletions obtained in northern China. In particular, we detected significant correlation between preS deletion and antiviral therapy. We also investigated whether preS deletion mutants were resistant to antiviral drugs based on an *in vitro* assay.

## Results

### Deletion patterns in HBV genomes prevailing in northern China

Full-length sequences were obtained from 51 patients including 38 males and 13 females with a mean age of 38.2 ± 13.1 years. Among these, 12 were genotype B and 39 were genotype C (Table 1).

**Table 1:** Clinical information of the LC/HCC group and the CC/CH group

**Features**	**CC%CH**	**LC%HCC**	**P value**
*Count*	33	18	-
*Antiviral Therapy*	14 (42%)	3 (17%)	-
*Age (mean ± SD)*	33 ± 10	49 ± 12	<0.001
*Gender (male%)*	24 (73%)	14 (78%)	0.483
*Genotype(C/B)*	23/10	11/7	0.375
*HBV-DNA > 10*^*7*^*copies/ml*	23 (70%)	9 (50%)	0.139
*Deletion mutants*	13 (39%)	7 (39%)	0.606
*PreS deletion mutants*	6 (18%)	5 (28%)	0.325
*BCP deletion mutants*	8 (24%)	3 (17%)	0.401

**Figure 1:**
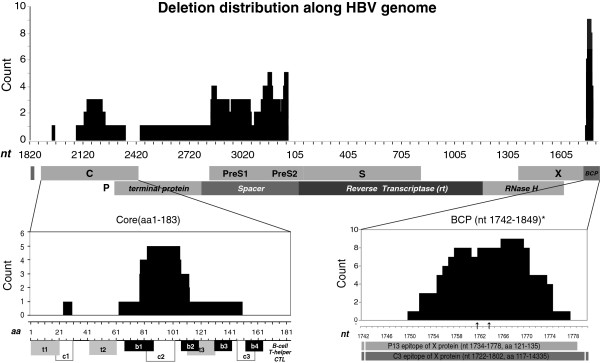
**Genome-wide deletion distribution of HBV in northern China. Upper panel:** The nucleotide location of deletions along the viral genome (X axis) and their counts (Y axis) in deletion mutations resolved from 51 whole genome sequences. Numbers at X indicate nucleotide position with the EcoR1 site at the preS1 region as 0. **Middle panel**: The ORFs for all genes, 4 domains of the P gene, and the BCP region. **Bottom Panel:** Alignment of detected deletions with viral epitopes in C (left) and the BCP/X region (right). 3 core deletions identified in clone sequencing were also included in addition to 4 deletions observed in whole genome sequences. The two arrows (bottom right) stand for nt 1762 and 1764 position, respectively. Known B- and T-cell epitopes in the C protein [35] are numbered from N- to C-terminus.

Of these 51 samples, genomic deletions were detected in 39% (20/51). As shown in Figure 1A, the deletions occurred almost exclusively in C, preS, and BCP regions with lengths varying from 2 to 496 nt, whereas no deletions were observed in the S gene, encoding the small surface protein.

Next we analyzed deletion boundaries from all full-length sequences. PreS deletions often occur around nt 2848-3215-56, whereas the C gene and BCP region tend to lose nt 2148–2219 and nt 1758–1770, respectively (Figure 1B-C). Deletion lengths in the BCP regions appeared consistently in two patterns as either 8-10bp (5/12) or 19-21bp (6/12).

### The influence of deletions on viral proteins and the BCP region

Of the three hotspots examined above, most deletions in X/BCP (12/14) and the C gene (4/7) were frameshift deletions, but almost all deletions in the preS (82/86) were in-frame deletions. In addition, the P gene related deletions never occurred at the reverse transcriptase region, which is the target of all nucleos(t)ide analogues (Figure 1A). The longest deletion (nt 2448–2934) shortened the polymerase by a third and removed most of the spacer and terminal protein domains.

**Figure 2:**
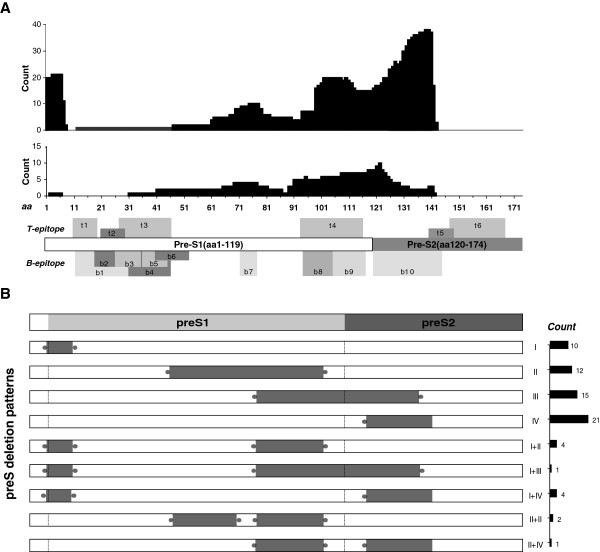
**Fine mapping of preS deletions. A.** Alignment of detected preS deletions in HBV spreading in northern China (upper panel) with the mutations in the same region from 6 immune-suppressed kidney-transplant patients from a previous study (middle panel) [4]. Known B- and T-cell epitopes in the preS region [18] are numbered from N- to C-terminus. Note the dramatic difference in deletion break points of preS2 and the higher deletion frequency at the 5^′^ terminus of preS1 between the two sample groups. The T- and B-cell epitopes of surface proteins are indicated in the bottom panel. **B.** PreS deletion patterns and their frequencies (right bars in black) in HBV prevailing in northern China. Sorting of 70 mutant clones resulted in four single patterns (I-IV) and four complex patterns as type I, start codon defect of L protein; type II, internal deletion of preS1; type III, start codon defect of M protein; and type IV, internal deletion of preS2. Gray bars indicate deletion positions. Blunt terminuses illustrate consistent break points and dotted edges display variable ends of deletions. Dashed lines show start codons in preS1 and preS2. Bars in black, right panel: The counts of different deletion patterns.

The most significant consequence of sequence deletion is the change of viral epitopes, in the core gene, the majority of deletions altered epitopes of the C2 domain (aa 84–101) of cytotoxic T lymphocytes (CTL) and the B1 domain (aa 74–89) of B-cells (Figure 1B). As shown in Figure 1C, the most frequently deleted fragment of BCP also covered nt 1753–1769 encoding aa 127–133 of the X protein, which interrupted previously reported targets of HBxAg-specific humoral immune response P13 (aa121-135) and C3 (aa117-143) [22,23]. As illustrated in Figure 2A, deletions in preS tend to affect t4, b8, b9 and b10 epitopes. Interestingly, despite the fact that almost all internal deletions of preS1 were localized at the b7 epitope (aa 72–78), far less truncations were seen in the upstream region where most B- and T-cell epitopes were clustered. The deleted domain in preS2 mutations spanned the b10 epitope (aa 120–145) and a couple of amino acids of the t5 epitope (aa 140–149), leading to truncated MHBsAgs. Notably, in contrast with a previous study where immunosuppressed patients showed lower preS2 deletion frequency, truncated preS2 mutants were most frequently observed in patients with preS deletions in our cohort.

Furthermore, most deletions in BCP occurred in non-coding regions without interrupting the transcription initiation site (nt 1793) of precore mRNA. The frequently reported single point mutations at nt 1762 (A) and 1764 (G), known to affect binding of BCP to liver-specific transcription factors that consequently reduce HBeAg expression, were included in most BCP deletions (10/14) (Figure 1C). However, patients with truncated BCP were actually HBeAg-positive, implying the co-existence of *wt* strains and possible coordination between *wt* and deletion mutants as evidenced by the existence of ~30% of *wt* detected during clone sequencing.

### Substructure of PreS deletions

As demonstrated in Figure 2A, the length and position of deletions in preS exhibited very diverse patterns. To explore the structural features of these mutations, we further amplified the preS region from a second cohort of 52 individuals and 70 preS deletions were obtained in clone sequencing. These truncations can be grouped into four categories, including a start codon defect of the L protein (I), an internal deletion of preS1 (II), a start codon defect of the M protein (III), an internal deletion of preS2 (IV), and complex patterns containing more than one deletion type (Figure 2B). Among these mutants, internal deletions of preS2 were the most common (37%, 26/70). Furthermore, nearly half (9/19) of the strains with type I deletions lost the same fragment (nt2848-2865). Also, more than half (9/16) of type III deletions were identical, with a 129 bp truncation at nt 3111-3215-24 disrupting the t4 and b9-10 epitopes (Figure 2A). Particularly, preS2 deletions had a variable 5^′^ terminus and fixed 3^′^ end (nt 54 to nt 56).

Type I and III deletions (34/70) also interrupted the start codons of surface proteins, leading to abolishment of LHBsAg or MHBsAg in 53% (18/34) and 44% (15/34) of cases respectively, with the remaining case (1/34) showing a complex deletion pattern, resulting in the loss of both antigens. In addition, we also detected a single base mutation, ATG to ATA, in preS2 from deletion mutants (5/70), resulting in the inability to produce M protein instead of making a truncated one. Therefore, both substitutions (5/70) and deletions (16/70) at the start codon led to the total abolishment of M protein production in 30% (21/70) of cases.

### Correlation of deletions with antiviral treatment

Next, we investigated a possible correlation between antiviral treatment and deletion patterns and analyzed clinical data of all dominant strains of quasispecies (Table 1). Logistic regression analysis illustrated the relationship between deletions and clinical factors including age, gender, diagnoses, genotypes, HBV DNA titers, and antiviral medication. Among all clinical factors examined, as summarized in Table 2, only antiviral treatment played a role in the accumulation of deletion mutations (Odds ratio [OR] = 6.81, 95% confidence interval [CI] = 1.296 ~ 35.817, P = 0.023). In addition, as shown in Table 1, we did not observe a higher overall deletion rate in advanced liver diseases (LC and HCC) as reported by other studies, possibly due to limited cases of HCC.

**Table 2:** The correlation between host factors and the occurrence of deletions by logistic regression analysis

**Predictor**	**B**	**S. E.**	**Wald χ**^***2***^	***p***	**OR**	**95.0% CI**	
						***Lower***	***Upper***
*Age*	0.016	0.035	0.21	0.646	1.016	0.948	1.089
*LogHBV_DNA*	0.075	0.328	0.052	0.819	1.078	0. 567	2.051
*Gender (Male/female)*	−0.534	0.766	0.487	0.485	0.586	0.131	2.629
*Group (LC%HCC/CC%CH)*	0.257	0.986	0.068	0.794	1.293	0.187	8.928
*Genotype (C/B)*	−0.351	0.83	0.179	0.672	0.704	0.138	3.577
*Antiviral Therapy (Treated/untreated)*	**1.919**	**0.847**	**5.138**	0.023*	**6.814**	**1.296**	**35.817**

B, regression coefficient; S.E., Standard Error; *, P < 0.05; OR, odds ratio; CI, confidence interval.

**Figure 3:**
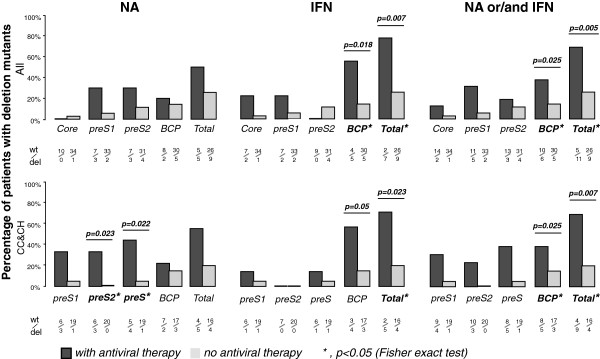
**Deletion mutations and antiviral treatments.** The profiles of different types of HBV deletions between patients with (+) and without (−) antiviral therapy based on 51 HBV full-length sequences. Upper panel: analysis from all samples of CC, CH, LC, and HCC. Lower panel: analysis without patients of progressive liver diseases. Antiviral medication was grouped as nucleotide analog only (left), IFN only (middle), and either one or both (right). Each deletion and the ratio of *wt* virus to mutants were labeled under the histogram.

Further comparison between sample groups also demonstrated that individuals with antiviral therapy showed a higher occurrence of deletions compared to the untreated group (P = 0.005, FET, Figure 3). A similar result was seen when the analysis was applied only to chronic carrier (CC) and chronic hepatitis (CH) patients (P = 0.007, fisher exact test (FET), Figure 3) when the possible contribution of mutant accumulation in advanced liver diseases was removed. When stratifying each deletion hotspot by antiviral therapy, BCP deletions were more common in patients with interferon therapy (P = 0.018, FET Figure 3), whereas deletions in preS, particularly in the preS2 region, were more likely to be found in cases with nucleotide analog (NA) treatment (P = 0.023, FET, Figure 3). In addition, sequencing data of the preS clones from the second batch of 52 individuals support the full-length analysis results. Of 10 CH patients containing preS2-deleted viruses detected by clone sequencing, 5 had received NA treatment, while 2 were treated with Interferon-alpha (IFN-α). Meanwhile, no significant difference in deletion occurrence was found between different genders (P = 0.608, FET) or genotypes (P = 0.450, FET). In addition, two out of three preS2 deletion mutants in the antiviral group had known antiviral resistance mutations, M204I and L180M, respectively.

### Dynamic accumulation of preS deletion mutants in HBV quasispecies during ADV treatment

The above results suggest that NAs may contribute to the accumulation of preS deletion mutants in quasispecies of CH patients. To further verify NAs’ selection in this region, we collected blood samples from two CH patients before and after about 3 months of ADV treatment. Serial samples were also collected from additional CH and LC patients, in intervals of 2.5 months and 5 months respectively, with no-antiviral treatment as the control. PCR direct sequencing of the preS region showed heterogeneous indels in the two samples collected after ADV treatment, whereas no deletion signal was seen before treatment or in the no-treatment controls. Furthermore, clone sequencing was performed in two samples showing heterogeneous indels. As demonstrated in Table 3, quasispecies analysis indicates that about half of the strains contain preS deletions in these two patients.

**Table 3:** Occurrence of preS deletion mutants in serial samples during ADV treatment

**Patients (CH)**		**Start**	**End**	
		***PCR direct sequencing***	***PCR direct sequencing***	***Clone sequencing***
**ADV**	1	N	D	aa 65–78 (peS1),
**(+)**		(Jan 24, 2005)	(Mar 22, 2005)	3/5 clones
	2	N	D	aa 132–141 (preS2),
		(Dec 15, 2004)	(Mar 21, 2005)	2/5 clones
**ADV**	3	N	N	-
**(−)**		(Dec 17, 2004)	(Feb 28, 2005)	
	4	N	N	-
		(Jan 14, 2005)	(Jun 7, 2005)	

N, no deletion detected; D, deletion detected.

### No antiviral resistance resulted from preS2 deletion alone

**Figure 4:**
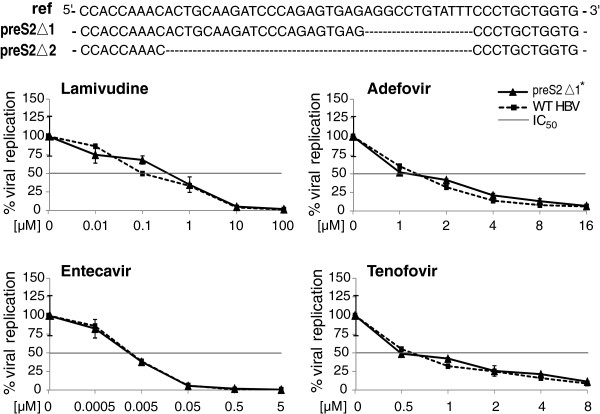
**Constructed preS2 mutants and their sensitivity to antiviral drugs.** Two deletions illustrated at the top were introduced into the *wt* genome in a plasmid, respectively. Constructed mutants were transfected into Huh7 cells with or without antiviral drug treatment as indicated in each plot. The viral replication level in a culture medium without drugs was denoted as 100%. The curves indicate the decrease in viral replication with increasing drug concentrations and the preS2 deletion alone did not change the mutants’ sensitivity to antiviral drugs. The crossover points between the horizontal line and the curves indicate the IC_50_ for each strain. *similar viral replication data of the Δ2 mutant with drug treatment is shown in Additional file 1: Figure S1.

Next, we investigated if deletions alone could directly lead to antiviral resistance. Two preS2 deletions with high occurrence rates were introduced into the *wt* strain in a plasmid followed by treatment with lamivudine, adefovir, entecavir and tenofovir. As shown in Figure 4 and Additional file 1: Figure S1A-D, both preS2Δ1 and preS2Δ2 showed similar sensitivity to the *wt* strain for all four drugs. Since the *wt* strain in the plasmid was genotype D whereas our data were mainly from genotype C strains, we further tested a similar preS2 mutant using the genotype C plasmid and obtained the same result (data not shown). Therefore, these preS2 deletion mutants alone did not have antiviral resistance.

We further compared the replication abilities of these strains in the absence of antiviral drugs, using HBsAg as the internal standard. Compared to the *wt* strain (100%), both mutants demonstrated slightly higher replication capacities (preS2Δ1,117%; preS2Δ2, 107%), however, statistical significance was not reached (Additional file 1: Figure S1E).

## Discussion

### Deletion patterns in the preS region upon host response to viral infection

We analyzed deletions in HBV genomes with respect to deletion hotspots and their boundaries, the correlation of mutations to antiviral medication, and the structural features in preS deletions. We compared preS deletions in our samples with those in immuno-suppressed patients reported by Preikschat *et al*. [4]. Similar deletion patterns in C, BCP, and preS1 regions were observed and one of the major differences was the deletion frequency in preS2 (Figure 2A). Among all deletions in the entire preS region, truncations in preS2 were the most common in our investigation, suggesting that the preS2 region may be selectively affected by immune pressure. Further studies are needed to clarify the role played by the host immune response in inducing deletions in preS1 and preS2 genes*.* Another interesting phenomenon is the high rate of deletions in the 5^′^ terminus of preS1 in our samples compared to immune-suppressed subjects as shown in Figure 2A. Although it does not encode any known epitope, this region spans the host determining region which contributes to the species specificity of HBV [24]. Interestingly, genotype D of HBV, which is 11 amino acids shorter than that of genotype B, does not contain this region and resembles the 5^′^ terminus of the preS1 deletion mutant [25].

### PreS2 deletions may promote HBV immune escape after recovery of host immune function following antiviral treatment

Deletions have been shown to confer resistance to lamivudine (LMV) in an HIV-related study, and certain deletion mutants of HBV were shown to be insensitive to LMV [26,27]. In our study, we observed the accumulation of preS deletions correlating to antiviral therapy. However, our *in vitro* experiments demonstrated that the HBV with preS deletion alone did not confer resistance to antiviral therapy in such mutants, similar to a recent observation by Ohkawa et al. [28].

This inconsistency between the epidemiological statistics and *in vitro* experiments is perhaps not surprising when the most common feature of HBV infection, the existence of quasispecies within an individual, is considered. Despite very complex patterns of HBV quasispecies, which were resolved by clone sequencing or high throughput sequencing, we, along with others, have observed that the wild type never disappears from the viral composition. For instance, our recent pyrosequencing study on HBV quasispecies showed that the lowest proportion of the *wt* strain in patients was around 1% (Zhang et al., unpublished). These data strongly suggest the coordination of *wt* and various types of mutants which may not survive by themselves alone but whose presence may be beneficial to the viral population *in vivo*.

Such coordination between viral strains may well explain our results. Generally speaking, antiviral therapy would also result in the recovery or enhancement of the host defense system, which in turn would increase the selection pressure on mutants, such as preS deletions, that may promote immune escape. Supporting evidence also stems from research that suggests an improvement in CTL responsiveness to HBV in CH patients following LMV treatment [29]. In addition, conventional vaccination combined with clevudine could help the host to overcome immunologic tolerance and restore T-cell responses to surface proteins according to the woodchuck model [30]. For instance, viruses with truncated or abolished M protein may survive due to the disruption of their epitopes. Interestingly, we observed a much higher frequency of preS2 deletions in patients treated with NAs compared to long-term immuno-suppressed organ-transplant recipients (Figure 2), suggesting increased immune escape in preS2 deletion mutants. In particular, almost all truncated preS2 mutants had a damaged b10 epitope (aa 120–145), a major envelope epitope whose absence would inhibit HBV clearing by the host [31,32]. Therefore, larger sample sizes and detailed functional analysis will be required for further verification. Meanwhile, considering the virulent feature of preS deletion mutants in chronic hepatitis infection, development of diagnostic tests for various deletion mutants would be beneficial for CH patients.

## Conclusions

In this study, we characterized deletion patterns in three hotspots, along the whole HBV genome, that are prevalent in northern China. Except for the BCP region, which influences regulating elements of the core gene, most deletions appear to destroy various epitopes of viral proteins. A comparison of samples with or without antiviral medication demonstrated a correlation between NA treatment and preS deletions, which is also evidenced by the analysis of serial samples before and after ADV treatment. Although preS deletions alone had no effect on drug resistance, the accumulation of preS deletion mutants in patients during antiviral treatment may promote viral immune escape*.*

## Methods

### Patients and blood samples

Blood samples were provided by You’an Hospital in Beijing. This study was approved by the Institutional Review Board of the Beijing Institute of Genomics and the Ethics Committee of Beijing You’an Hospital of Capital Medical University. Informed consent was obtained from all patients. Patients were diagnosed as chronic carrier (CC), chronic hepatitis (CH), liver cirrhosis (LC) and hepatocellular carcinoma (HCC) according to the guidelines on the prevention and treatment of chronic hepatitis B in China (2010) [33]. No patients had co-infections with HCV, HDV, or HIV. Blood samples of 5ml were collected, cells and sera were then separated and stored at −20°C. From the few hundred stored samples, we successfully amplified and sequenced 51 whole genomes from 51 individuals. Additionally, preS clone sequencing was performed in another cohort of 52 patients for fine mapping of deletion substructure.

### DNA quantification and HBV serological marker detection

Viral DNA titers were quantified using the FQ-PCR Kit for HBV (DaAn Gene Co., Guangdong, China) on a GeneAmp 5700 Sequence Detection System (PE Applied Biosystems, CA, USA). Serological markers were determined by an Electrochemiluminescence Immunoassay on a Roche E170 Modular Immunoassay Analyzer (Roche Diagnostics, Mannheim, Germany) following the manufacturer’s protocol.

### Viral DNA extraction and amplification

Viral DNA was extracted from 400 μl sera per sample using the QIAamp MinElute Virus Spin (Qiagen, Hilden, Germany). All DNA samples were stored at −20°C.

Whole genome amplification was performed using LA Taq (Takara, Osaka, Japan) according to the method described by Günther et al., with the primers for P1(1821 to 1841), CCGGAAAGCTTGAGCTCTTCTTTTTCACCTCTGCCTAATCA,  and  P2  (1823 to 1806),  CCGGAAAGCTTGAGCTCTTCAAAAAGTTGCATGGTGCTGG [34]. The lowest DNA amount required for amplification was 10^3^ copies/ml in our experimental system. Sequencing primers are listed in Additional file 1: Table S1, and the primers SP5 and SP9 were also used for preS region amplification. Hot start PCR for the preS region was performed with the following cycle: 95°C for 2 minutes and 30 seconds, followed by 35 cycles of denaturation at 94°C for 1 minute, annealing at 58°C for 90 seconds, and elongation at 72°C for 3 minutes. All reactions were performed on a PTC-200 Peltier Thermal Cycler (MJ Research, MA, USA).

### Viral DNA sequencing

After purification via the Montage PCR96 column (Millipore, MA, USA), PCR products were sequenced on a Prism 3730 (ABI, USA). Contigs were assembled using SeqMan (DNAstar 5.0, WI, USA), and sequences were aligned using ClustalW for further analysis. All mutations were checked manually. Whole genomes mentioned in this study are defined as >97% of full length and sequencing gaps at the end of the genome have no overlaps with deletion hotspots.

The boundaries of deletion regions that appeared in the sequencing electropherogram were determined by reading from both directions. The regions of interest were amplified by PCR and the products were cloned into a pMD18 T vector (Takara, Osaka, Japan) followed by sequencing of 5–10 positive clones per sample. NCBI accession numbers for all sequences are listed in Additional file 1: Table S2.

### Construction of HBV mutants and examination of their antiviral resistance

Candidate deletions were introduced into the HBV-expression plasmid Yi026-pcDNA3.1/Zeo(−) using the QuikChange® Site-Directed Mutagenesis Kit (Stratagene, CA, USA). The plasmid, harboring a 1.1X overlength genome of HBV (*ayw*), was kindly provided by Yi Ni and Stephan Urban from the University of Heidelberg (Heidelberg, Germany). Introduced mutations were verified by plasmid re-sequencing.

HuH7 cells were seeded into 10 cm^2^ dishes at 1.5 × 10^6^ cells/dish, reaching around 90% confluency before transfection the following day. Cells were transfected using 24 μl FuGENE®HD (Roche, IN, USA)) reagent with 8 μg of plasmid DNA. 16–20 h post-transfection, transfected cells were washed twice and then seeded into a 96-well plate at 3 × 10^4^ cells/well. The cells were treated with serial dilutions of four drugs in fresh medium for 3 days, including lamivudine (LMV), adefovir (ADV), entecavir and tenofovir (Sequoia Research Products Limited, UK). The supernatants were collected after centrifugation at 1500 × g for 5 min, and then prepared for HBV DNA extraction using the BioSprint 96 One-For-All Vet Kit (Qiagen, Hilden, Germany). Half maximal inhibitory concentrations (IC_50_) were calculated for each construct where the resistance factor is calculated as the IC_50_ of mutant divided by the IC_50_ of the *wt* strain. The amount of HBsAg produced by each strain was determined by the AxSYM HBsAg assay (Abbott Laboratories, IL, USA).

### Statistical analysis

SPSS 13.0 was used for logistic regression analysis, t-tests and Fisher exact tests (FET).

## Competing interests

The authors declare that they have no competing interests.

## Authors’ contributions

DZ performed the original data analysis. PD and HD collected samples and did clinical data analysis. LD, WC, and FL took part in sequencing experiments and data analysis. In vitro experiments were designed and performed by KZ, CB and UP. HD and CZ guided and designed the project. DZ and CZ prepared the bulk of the manuscript. All the authors read and approved the final manuscript.

## Supplementary Material

Additional files 1 **Figure S1.** Antiviral resistance examination for the preS2Δ2 mutant. Table S1. Primer sequences. Table S2. Accession numbers for nucleotide sequences. Click here for file
