# Supplementary material for: Whole genome HBV deletion profiles and the accumulation of preS deletion mutant during antiviral treatment
Source: BMC Microbiol. 2012 Dec 28;12:307. doi: 10.1186/1471-2180-12-307 (PMC3549285; doi:10.1186/1471-2180-12-307)
Supplement: Additional files 1 — Figure S1. Antiviral resistance examination for the preS2Δ2 mutant. Table S1. Primer sequences. Table S2. Accession numbers for nucleotide sequences. [file 1471-2180-12-307-S1.doc]

**­­Supplementary**

**Fig.S1 Antiviral resistance examination for the preS2△2 mutant.** Constructed mutants were transfected into Huh7 cells with or without antiviral drug treatment as indicated in each plot. The viral replication level in a culture medium without drugs was denoted as the level of 100%. The curves in A-D indicated the decrease of viral replication along with the increase of drug concentration and preS2 deletion alone did not change the mutants’ sensitivity to antiviral drugs. The crossover points between the horizontal line and the curves indicated IC_50_ for each strain. E. Replication capacity test for 3 strains.

**A B**

**
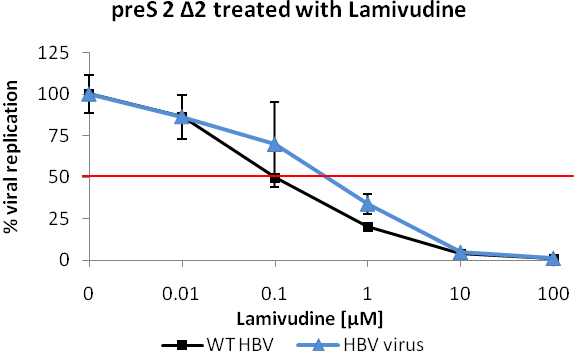

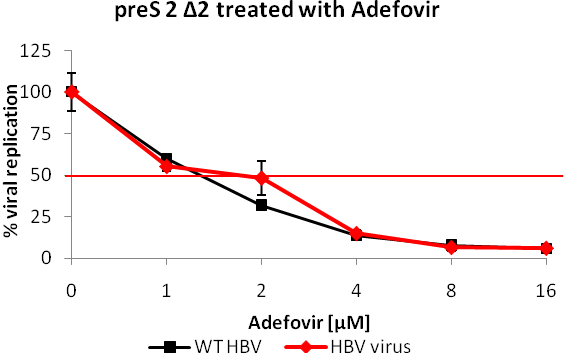
**

**C D**

**
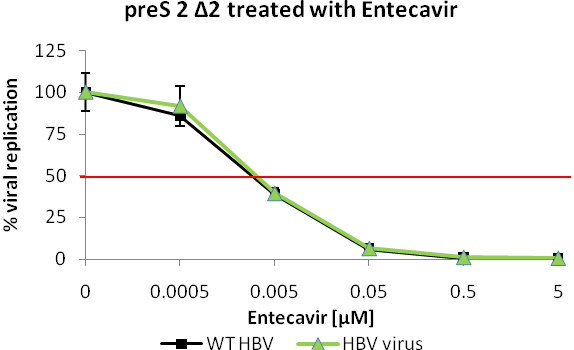

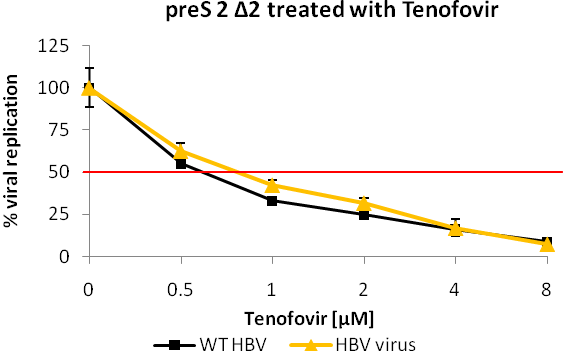
**

**E**


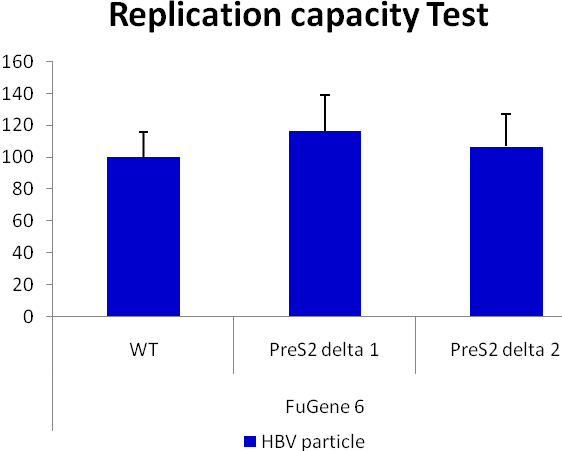


**Table S1. Primer Lists**

| ***Primer*** | ***Nucleotide Sequence*** | ***Position*** | ***Usage*** | ***Polarity*** |
| --- | --- | --- | --- | --- |
| **SP1** | ***5'***TTTTTCACCTCTGCCTAATCA | 1821-1841 | seuquencing | + |
| **SP2** | ***5'***AGTTCTTCTTCTAGGGGACCTGCC | 2357-2380 | sequencing | - |
| **SP3** | ***5'***CGCAGAAGATCTCAATCTCGG | 2417-2437 | sequencing | + |
| **SP4** | ***5'***GGATAGAACCTAGCAGGCAT | 2637-2656 | sequencing | - |
| **SP5** | ***5'***TCACCATATTCTTGGGAACAAGA | 2817-2839 | preS amplification/sequencing | + |
| **SP6** | ***5'***GAACTGGAGCCACCAGCAGG | 56-75 | sequencing | - |
| **SP7** | ***5'***CTCCAGTTCAGGAACAGTAAACCC | 67-90 | sequencing | + |
| **SP8** | ***5'***ATTCCTATGGGAGTGGGCCTCAG | 634-656 | sequencing | + |
| **SP9** | ***5'***CGAACCACTGAACAAATGGC | 685-704 | preS amplification/sequencing | - |
| **SP10** | ***5'***CCATACTGCGGAACTCCTAGC | 1266-1286 | sequencing | + |
| **SP11** | ***5'***GAGACCACCGTGAACGCCCA | 1611-1630 | sequencing | + |

**Table S2. Accession numbers for nucleotide sequences.**

| 51 HBV whole genomes | | | | | | |
| --- | --- | --- | --- | --- | --- | --- |
| GenBank ID: JQ040125 | GenBank ID: JQ040135 | GenBank ID: JQ040145 | GenBank ID: JQ040155 | GenBank ID: JQ040165 | GenBank ID: JQ040175 |  |
| GenBank ID: JQ040126 | GenBank ID: JQ040136 | GenBank ID: JQ040146 | GenBank ID: JQ040156 | GenBank ID: JQ040166 |  |  |
| GenBank ID: JQ040127 | GenBank ID: JQ040137 | GenBank ID: JQ040147 | GenBank ID: JQ040157 | GenBank ID: JQ040167 |  |  |
| GenBank ID: JQ040128 | GenBank ID: JQ040138 | GenBank ID: JQ040148 | GenBank ID: JQ040158 | GenBank ID: JQ040168 |  |  |
| GenBank ID: JQ040129 | GenBank ID: JQ040139 | GenBank ID: JQ040149 | GenBank ID: JQ040159 | GenBank ID: JQ040169 |  |  |
| GenBank ID: JQ040130 | GenBank ID: JQ040140 | GenBank ID: JQ040150 | GenBank ID: JQ040160 | GenBank ID: JQ040170 |  |  |
| GenBank ID: JQ040131 | GenBank ID: JQ040141 | GenBank ID: JQ040151 | GenBank ID: JQ040161 | GenBank ID: JQ040171 |  |  |
| GenBank ID: JQ040132 | GenBank ID: JQ040142 | GenBank ID: JQ040152 | GenBank ID: JQ040162 | GenBank ID: JQ040172 |  |  |
| GenBank ID: JQ040133 | GenBank ID: JQ040143 | GenBank ID: JQ040153 | GenBank ID: JQ040163 | GenBank ID: JQ040173 |  |  |
| GenBank ID: JQ040134 | GenBank ID: JQ040144 | GenBank ID: JQ040154 | GenBank ID: JQ040164 | GenBank ID: JQ040174 |  |  |
| 70 cloned preS sequences | | | | | | |
| GenBank ID: JQ040176 | GenBank ID: JQ040186 | GenBank ID: JQ040196 | GenBank ID: JQ040206 | GenBank ID: JQ040216 | GenBank ID: JQ040226 | GenBank ID: JQ040236 |
| GenBank ID: JQ040177 | GenBank ID: JQ040187 | GenBank ID: JQ040197 | GenBank ID: JQ040207 | GenBank ID: JQ040217 | GenBank ID: JQ040227 | GenBank ID: JQ040237 |
| GenBank ID: JQ040178 | GenBank ID: JQ040188 | GenBank ID: JQ040198 | GenBank ID: JQ040208 | GenBank ID: JQ040218 | GenBank ID: JQ040228 | GenBank ID: JQ040238 |
| GenBank ID: JQ040179 | GenBank ID: JQ040189 | GenBank ID: JQ040199 | GenBank ID: JQ040209 | GenBank ID: JQ040219 | GenBank ID: JQ040229 | GenBank ID: JQ040239 |
| GenBank ID: JQ040180 | GenBank ID: JQ040190 | GenBank ID: JQ040200 | GenBank ID: JQ040210 | GenBank ID: JQ040220 | GenBank ID: JQ040230 | GenBank ID: JQ040240 |
| GenBank ID: JQ040181 | GenBank ID: JQ040191 | GenBank ID: JQ040201 | GenBank ID: JQ040211 | GenBank ID: JQ040221 | GenBank ID: JQ040231 | GenBank ID: JQ040241 |
| GenBank ID: JQ040182 | GenBank ID: JQ040192 | GenBank ID: JQ040202 | GenBank ID: JQ040212 | GenBank ID: JQ040222 | GenBank ID: JQ040232 | GenBank ID: JQ040242 |
| GenBank ID: JQ040183 | GenBank ID: JQ040193 | GenBank ID: JQ040203 | GenBank ID: JQ040213 | GenBank ID: JQ040223 | GenBank ID: JQ040233 | GenBank ID: JQ040243 |
| GenBank ID: JQ040184 | GenBank ID: JQ040194 | GenBank ID: JQ040204 | GenBank ID: JQ040214 | GenBank ID: JQ040224 | GenBank ID: JQ040234 | GenBank ID: JQ040244 |
| GenBank ID: JQ040185 | GenBank ID: JQ040195 | GenBank ID: JQ040205 | GenBank ID: JQ040215 | GenBank ID: JQ040225 | GenBank ID: JQ040235 | GenBank ID: JQ040245 |
